# Regeneration and growth in crowns and rhizome fragments of Japanese knotweed (*Reynoutria japonica*) and desiccation as a potential control strategy

**DOI:** 10.7717/peerj.11783

**Published:** 2021-08-12

**Authors:** Jacob W. Lawson, Mark Fennell, Mark W. Smith, Karen L. Bacon

**Affiliations:** 1 School of Geography, University of Leeds, Leeds, United Kingdom; 2 AECOM, Cambridge, United Kingdom; 3 Botany and Plant Science, National University of Ireland, Galway, Galway, Ireland

**Keywords:** Japanese knotweed, Regeneration, Nodes, Crowns, Rhizomes, Desiccation, Management, Invasive species

## Abstract

*Reynoutria japonica* (Japanese knotweed) is a problematic invasive plant found in many areas of Europe and North America. Notably, in the UK, the species can cause issues with mortgage acquisition. Control of *R. japonica* is complicated by its ability to regenerate from small fragments of plant material; however, there remains uncertainty about how much (in terms of mass) rhizome is required for successful regeneration. This study investigated the ability of crowns and rhizomes with different numbers of nodes to regenerate successfully from three sites in the north of England, UK. Two of the sites had been subject to herbicide treatment for two years prior to sampling and the third site had no history of herbicide treatment. No significant differences were observed in regenerated stem diameter, maximum height of stem and maximum growth increments among crowns. All traits measured from the planted crowns were significantly greater than those of the planted rhizome fragments and at least one node was necessary for successful regeneration of rhizomes. The smallest initial fragment weight to regenerate and survive the experiment was 0.5 g. Subjecting all plant material to desiccation for 38 days resulted in no regrowth (emergence or regeneration) after replanting. These findings suggest that desiccation could be a valuable management strategy for small to medium scale infestations common in urban settings.

## Introduction

**Figure 1:**
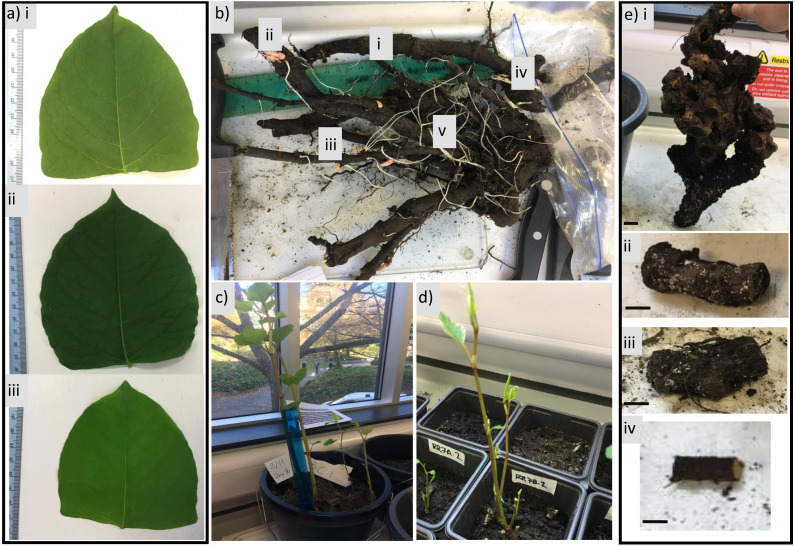
Examples of *R. japonica* material used in this study. (A) Example leaves from each site: (i) Burnley, (ii) Rodley, Leeds, (iii) Huddersfield; (B) rhizome network with examples of (i) dark woody rhizome; (ii) immature rhizome; (iii) pink buds; (iv) immature fleshy rhizome; (v) thin root; (C) example of lab-grown stems from field-collected crown; (D) examples of lab-grown stems beginning to emerge from field-collected rhizome fragments; (E) examples of field-collected plant fragment categories: (i) crown; (ii) rhizome fragment with two nodes; (iii) rhizome fragment with one node (iv); rhizome fragment with zero nodes; black bar = one cm.

*Reynoutria japonica* (formally *Fallopia japonica* and commonly called Japanese knotweed) is an herbaceous perennial of the Polygonaceae family and native to East Asia, including China, Japan and Korea. It has large, shield-shaped leaves, large crowns and produces dense canes (Fig. 1). It is recognised as one of the most problematic non-native invasive species globally (Lowe et al., 2000; Pyšek et al., 2003; Duquette et al., 2015; Gillies, Clements & Grenz, 2016; Jones et al., 2018). Its ability to regenerate and spread through vegetative growth and fragmentation (Child & Wade, 2000) makes it a difficult invasive species to manage notwithstanding its lack of viable seeds in the UK, Ireland and elsewhere (Bailey, Bímová & Mandák, 2009; Engler, Abt & Buhk, 2011). Some studies also suggest that increasing global temperatures may support the spread of hybrids that do set seed (e.g., Groenevld, ois Belzile & Lavoie, 2014; Buhk & Thielsch, 2015), which is likely to increase the difficulties of controlling this species. *R. japonica* is often found on road verges and alongside railways (Beerling, Bailey & Conolly, 1994; Palmer, 1994; Child & Wade, 2000; Fennell, Wade & Bacon, 2018) but primarily invades disturbed ecosystems, such as riparian zones, resulting in increased flood risk (Booy, Wade & Roy, 2015), significant biodiversity alterations (Gerber et al., 2008; Aguilera et al., 2010; Gillies, Clements & Grenz, 2016) and other impacts.

Various management options are available for control of Japanese knotweed, from avoidance to excavation, with herbicide treatment being the optimal option under most scenarios (Environment Agency, 2016). Herbicide causes the least damage to the surrounding environment, reduces the risk of accidental spread (Beerling, 1990; Hagen & Dunwiddie, 2008; Hagner et al., 2019) and costs considerably less than excavation. Biological control, despite some promising trials (e.g., Fung et al., 2020) is not currently an available option. Jones et al. (2018) evaluated the use of phenologically-timed herbicide application for the control of *R. japonica* and highlighted that late season treatments with a glyphosate-based herbicide produce the best outcomes. However, following herbicide treatment there is a risk of regrowth, especially if the ground is disturbed and dormant, viable rhizomes are brought to the surface. As such, research into cost effective control methods that could reduce the potential for regrowth is needed.

In the UK, *R. japonica* spreads primarily through the regenerative growth of crowns and rhizomes. The rhizome network is particularly important because there is considerable plasticity in the root:shoot ratio depending on available nutrients (Hirose, 1987). The rhizomes are known to twist and turn more than many other species (Smith et al., 2007) and to grow through even small spaces. However, despite the reputation that rhizomes are associated with major structural damage, there is no evidence that they are any more damaging to built structures than other plants (Fennell, Wade & Bacon, 2018). The importance of *R. japonica* rhizomes for reproduction and spread can be highlighted by observations that only small fragments of rhizome are required for regeneration (Brock & Wade, 1992; Beerling, Bailey & Conolly, 1994; Francis, Riley & Hoggart, 2008); however, there is uncertainty in the literature regarding the minimum size of rhizome fragment that can regenerate successfully. Brock & Wade (1992) observed regeneration in fragments as small as 0.7 grams, and this figure has gained sustained traction throughout the literature (e.g., Bailey, Bímová & Mandák, 2009). Other work has demonstrated a dependence of regeneration on rhizome length (Sásik & Pavol Jr, 2006) and Francis, Riley & Hoggart (2008) suggested that, for regeneration from stems, the number of nodes may be the most significant determinant. Overall, there is insufficient evidence to make conclusive statements on the relevant metric (rhizome weight, length or number of nodes) required for successful regeneration of rhizomes.

Stems, as well as rhizomes, are an organ of *R. japonica* that increase its invasive capabilities (Brock et al., 1995). With new stands growing each year and dead stems remaining upright over winter (Fig. 1) due to slow decay rates (Vrchotová et al., 2005), stem density (from both dead and alive stems) can achieve densities of 70 stems per m^−2^ (Callaghan, Scott & Whittaker, 1981), which increases the competitive advantage of *R. japonica* by utilising space, sunlight and resources (Townsend, 1997). Although this will vary due to environmental conditions and crown size, it is apparent that the combination of underground biomass (e.g., rhizome) behaviour and aboveground biomass structure (and density) facilitates *R. japonica* to outcompete native shrubs and woody perennials.

Management of *R. japonica* infested sites is complicated by the plant being classified as controlled waste in UK and Ireland when it is taken off a site, making disposing of it expensive. One aspect of potential management that has not been fully considered is desiccation. Although many plants are drought tolerant, far fewer are known to be fully desiccation tolerant. Drought tolerance usually means that plants are able to dry out to around 23% of their fresh weight with no bulk water present in their cytoplasm but desiccation tolerance refers to even further dehydration that —once rehydrated —does not damage the plant cells (Hoekstra, Golovina & Buitink, 2001). This anhydrobiosis is often an adaptation to severe environmental conditions. For example, the desert plant *Selaginella lepidophylla* commonly called the “resurrection plant” is known to be able to survive almost total desiccation. Although many mosses are desiccation tolerant, far fewer angiosperms and no gymnosperms are known to be able to survive desiccation (Alpert, 2005). Drying angiosperms to equilibrium with air (dry weight) kills virtually all of them (Alpert, 2006). Desiccation as a management technique for aquatic invasive non-native species has highlighted that such species can survive even significant drying (Coughlan et al., 2018), while terrestrial plants have been shown to be more susceptible to desiccation as a potential control method (e.g., *Rumex* species; Alshallash & Hatcher, 2020). However, no experiments focusing on how *R. japonica* responds to a period of air drying and replanting were identified in the literature.

This study assesses the following: (1) the regeneration of different sizes of *R. japonica* fragment both exposed and unexposed to herbicide treatment; (2) the effects of nodes on rhizome fragment regeneration; and (3) the effect of desiccation by air drying on rhizome fragment regeneration.

## Methods

### Fieldwork

**Figure 2:**
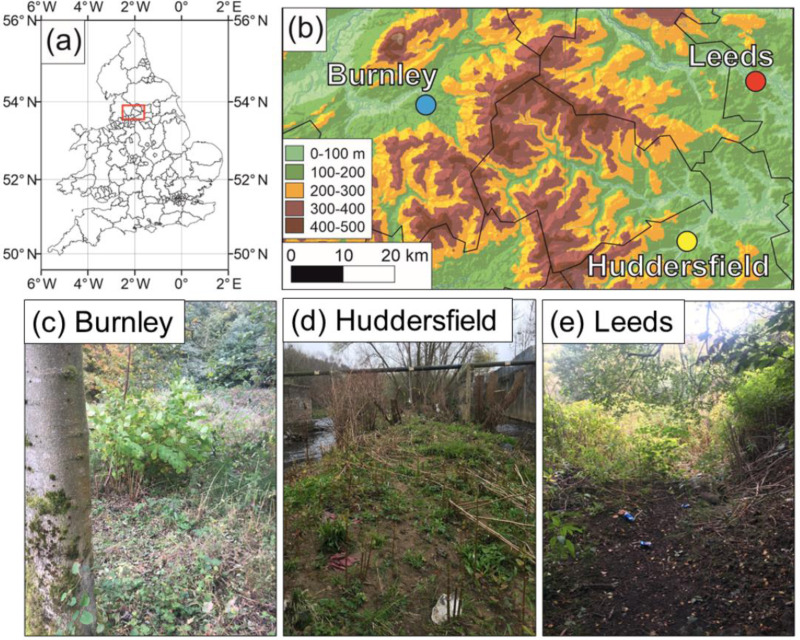
Field site locations in the United Kingdom. (A) Location of field sites in the UK; (B) close-up on the North of England locations of the sites (underlying DEM is the MERIT DEM (http://hydro.iis.u-tokyo.ac.jp/ yamadai/MERIT_DEM/) and waterways are in blue (https://mapcruzin.com/free-england-arcgis-maps-shapefiles.htm)) (C) Burnley field site; (D) Huddersfield field site; (E) Rodley, Leeds field site.

Three sites in the North of England, United Kingdom (Fig. 2), two with two years of herbicide treatment and one with no recorded treatment history, were sampled in early April 2019 near the start of the growing season, which avoided concerns about winter dormancy impacting results. Both treated sites (Burnley in Lancashire and Rodley, Leeds in West Yorkshire) were located by the side of the Leeds–Liverpool Canal, surrounded by a riparian habitat, with similar treatment histories (two years of glyphosate-based herbicide treatment applied in October 2017 and 2018). Both are managed by the Canal and Rivers Trust. The untreated site, Huddersfield, West Yorkshire, was similar though on an “island” in the Colne River, with slightly sandier soil and managed by the Yorkshire Wildlife Trust. Collection of plant material was undertaken between 1st and 4th April 2019. Ten crowns and three sections of rhizome per crown were excavated from each site using spade, trowel and hand (to gently remove the ends of the more delicate rhizomes). Crowns were selected randomly once they had at least one live cane emerging (current year’s growth) and one dead cane (showing that the rhizome had produced viable above-ground material in a previous year. Rhizomes were separated from the crowns at the point where they emerge from the crown. Once excavated, crowns and rhizomes were sealed in plastic bags and transported to the lab. Permission to sample was obtained in advance by the Canal and Rivers Trust and the Yorkshire Wildlife Trust respectively.

### Laboratory work

Once in the lab, crowns were fresh weighted and potted. Crowns varied in size as described in Supplemental Information. Plant fragments were potted in pots of an appropriate size based on fragment size. Rhizome fragments were potted in square sided pots of nine cm per side and crowns were potted in either medium-sized pots of 21 cm diameter or large-sized pots for crowns over 900 g fresh weight (five per site; Supplemental Information) of 30 cm diameter). Crowns were potted at surface level and rhizomes were covered with soil to a depth of approximately one cm. Plants were potted the day after collection and drying was prevented by storage overnight in a cold room. Since the composition of soil biota is a potential variable that could affect *R. japonica* growth (Parepa, Schaffner & Bossdorf, 2013), the soil mixture was standardised throughout the experiments, consisting of multi-purpose compost (John Innes Multi-Purpose compost) mixed with garden soil at a ratio of 1:4. The garden soil was sampled from an area in Wakefield, Leeds, that is known to not contain *R. japonica* and is a silty clay loam, similar to the sites where the samples were collected*.*

For the rhizome investigation, three segments of rhizome from each crown were collected, yielding a total of 30 segments from each site. One fragment with two nodes, one fragment with one node and one fragment with no nodes were sub-sampled from each rhizome segment resulting in three subsamples of each node classification from each crown, to reduce the possibility of one unhealthy rhizome distorting results. This resulted in the potting of 30 full crowns and 270 rhizome fragments in total.

All crowns and rhizomes were watered twice a week and the amount of water was normalised to the size of the pot (so that all samples received the same relative amount of water). Fertiliser (Baby Bio Original Plant Feed: standard NPK fertiliser with Nitrogen (10.6%); Phosphorus (1.9%), Potassium (1.4%) and organic matter) was added to the watering regime (10 ml per litre) on day 1, day 30 and day 50 of the 60-day experimental period to ensure the availability of nutrients throughout the experiment. Pots were randomly organised and placed on a laboratory bench by the window so that they were exposed to sunlight. They were subject to room temperature (with nightly minima above approximately 9 °C and daily maxima between 15–36 °C, with average daily temperature ranges of approximately 19–25 °C).

Both emergence (new growth) and regeneration were monitored throughout the experiment. Emergence was considered to have occurred when a new bud or stem was observed growing from the crown or rhizome fragment. Successful regeneration was considered to have occurred when a new bud emerged, burst and formed one leaf. Stem height was measured by recording the height from the surface of the soil to the tip of the stem. If stems did not show the traits mentioned, they were classed as an emergence but not regeneration.

Once the growth phase of the experiment ended (after 60 days), all underground biomass was left on plastic sheets to dry in the lab, which was generally between 19 and 25 °C during the day and around 9 °C minimum at night. Plant fragments were weighed at least once per week and this drying process continued until 10% or less of mass was lost between measurements and lasted for 38 days (by which time all plant fragments met these criteria including even the largest crowns (see Supplemental Information). The dried biomass was replanted under the same conditions as the first pot experiment and observed for an additional 60 day period. Treatment of the planted dry biomass replicated the initial 60 day growth experiment with fertiliser (Baby Bio) applied on day 1, 30 and 50 and water applied twice per week.

All field and laboratory work and the disposal of material at the end of the experiment followed strict biosecurity protocols in line with the University of Leeds health and safety standards.

### Statistical analysis

Anderson-Darling normality tests were completed prior to any test for differences in regeneration and growth rates between six categories (ANOVA or Kruskal-Wallis testing). Subsequent *post hoc* tests included Fisher’s pairwise comparison, Mann–Whitney U test or *T*-test depending on their distributions. A range of statistical software packages were used to obtain the results including R, Minitab (version 18) and GPower (Faul et al., 2009; GPower 3.1.9.4). ImageJ (2019, v1.51c: documentation and downloads at website http://rsbweb.nih.gov/ij/, National Institutes of Health, Bethesda, Maryland, USA) was used to measure leaf area. All raw data are provided in the Supplemental Information.

## Results

### Emergence and regeneration

**Figure 3:**
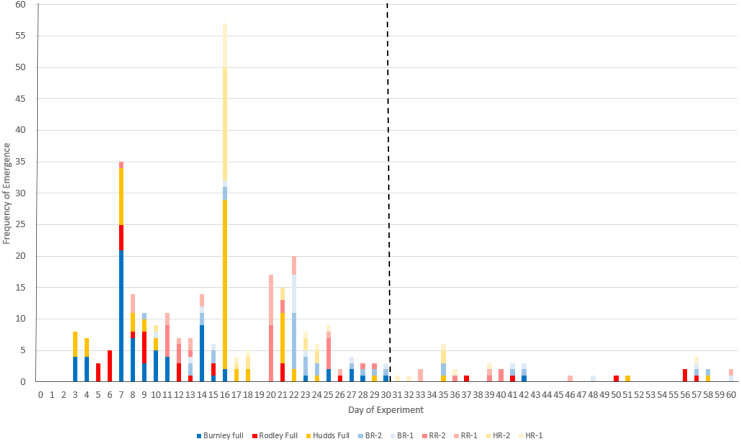
Frequency of new stem emergence per day of the 60-day experiment across all the categories of plant material. Colours refer to sites (Burnley in blue; Rodley in red and Huddersfield in yellow) and colour tones refer to size of plant material (darkest shades for full crown, medium shades for two node rhizome fragments and lightest tone for one node rhizome fragments). Full, full crown; number, number of nodes (1 or 2); BR, Burnley; RR, Rodley; HR, Huddersfield. The dashed line shows day 30.

The experiment ran for 60 days with 88.5% (293/331 stems) of stems emerging within the first 30 days (Fig. 3) and an additional 10 emerging during the final four days of the experiment. A total of 38 stems (11.48% of all regenerations) emerged between day 31 and day 60 (Fig. 3; Table 1), highlighting that 30 days in not long enough to track emergence (or regeneration) of *R. japonica*. All three sites had emergences during this period from both crowns and rhizomes.

**Table 1:** Regenerative success of all sites and size categories. Percentages rounded to nearest 0.1%.

** **	**NS/N**	**Success rate (%)**
**Burnley Full**	10/10	100%
**Burnley Rh 2**	20/30	66.7%
**Burnley Rh 1**	16/30	53.3%
**Burnley Rh 0**	0/30	0%
**Rodley Full**	8/10	80%
**Rodley Rh 2**	23/30	76.7%
**Rodley Rh 1**	24/30	80%
**Rodley Rh 0**	0/30	0%
**Huddersfield Full**	10/10	100%
**Huddersfield Rh 2**	25/30	83.3%
**Huddersfield Rh 1**	18/30	60%
**Huddersfield Rh 0**	0/30	0%

**Notes.**

Full full crowns Rh rhizomes number number of nodes NS number of rhizome fragments successfully regenerated from N, total number of samples

Day 16 had the most new emergences with 57 new buds emerging above the surface of the soil. Figure 3 shows that emergences from rhizomes experience a longer lag time compared to full crowns with the first crown emergences occurring on day 3 and the first rhizome emergences occurring on day 7.

Successful regeneration occurred in both crowns and rhizomes with one or two nodes. No emergence (or regeneration) was recorded from any rhizome fragment with zero nodes, regardless of fragment weight or length. Table 1 shows that full crowns exhibited the highest success rate with the only failed regenerations occurring in Rodley (20% (two crowns) failed to regenerate) and that with the increasing size categories (zero to one to two nodes to full crowns) there was an increase in successful regeneration.

### Growth

**Figure 4:**
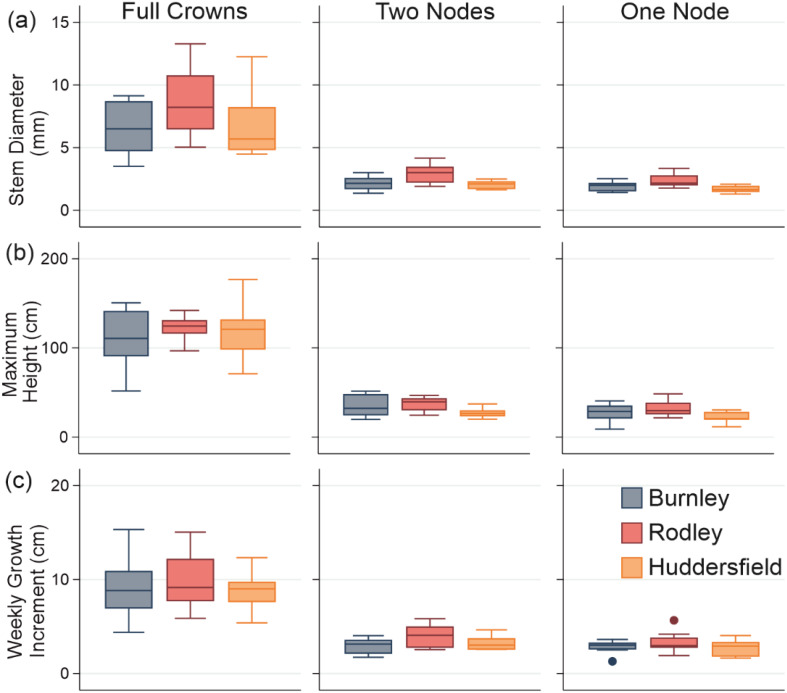
Stem growth across all categories of plant material from each site. (A) Diameter; (B) Maximum height; (C) Weekly growth increment. Columns show size categories: (i) full crowns; (ii) two nodes; (iii) one node. Colours refer to sites (Burnley in blue; Rodley in red and Huddersfield in yellow) and colour tones refer to size of plant material (darkest shades for full crown, medium shades for two node rhizome fragments and lightest tone for one node rhizome fragments) Boxes represent 75th (top of box) percentile to the 25th (bottom of box) percentile of the data with the median represented by the line between the two. Data ranges are represented by the whiskers.

Most of the substantial increase in height was observed in the first three weeks, with maximum growth usually occurring in weeks 2–4 (Fig. 4), for all plant fragment types but maximum growth was recorded for two Rodley one-node fragments and two Huddersfield crowns in week 5 and for one Rodley one-node fragment in week 6 (Supplemental Information). Thereafter, growth decreased or ceased towards the latter half of the study period. The highest weekly growth increment was 111.8 cm per week (Supplemental Information), by a Huddersfield full crown in its second week of growth. The minimum growth rate recorded was 0 cm for at least one plant fragment of each type .There was no significant difference between crowns (Kruskal-Wallis: *p* = 0.262, *H* = 2.68; Figs. 4B(i) and Figs. 4C(i)) or one node fragments (*p* = 0.206, *F* = 1.7; Figs. 4B(iii) and 4C(iii)) across the three sites. Two node rhizome fragments from Rodley produced stems with significantly higher growth than Burnley but Huddersfield showed no significance between the two (*p* = 0.056, *F* = 3.27; Fig. 4C(ii)). There was also a significant difference between full crowns and rhizomes at all three sites (Burnley: ANOVA, *p* < 0.0001, *F* = 32.25; Rodley: Kruskal-Wallis: *p* < 0.0001, *H* = 15.86; Huddersfield: ANOVA, *p* < 0.0001, *F* = 48.88) but no difference between one and two nodes for average weekly growth (Fig. 4).

The maximum heights of stems regenerating from full crowns showed no significant difference between sites (ANOVA: *p* = 0.575, *F* = 0.57; Fig. 4B(i)). There was also no significant difference between either the two-node or one-node stems between the sites (two-nodes: ANOVA; *p* = 0.584, *F* = 0.54; one-node: ANOVA; *p* = 0.166; *F* = 1.86; Fig. 4B(ii)). Stems that regenerated from crowns were significantly taller than stems that regenerated from rhizomes across all three sites (Burnley: *p* < 0.0001, *F* = 48.32, Rodley: *p* < 0.0001, *F* = 191.33, Huddersfield: *p* < 0.0001, *F* = 68.49, Fig. 4). There was also no significant difference in the maximum height between stems that regenerated from rhizome fragments with one or two nodes across each of the three sites (ANOVA; *p* = 0.567; *F* = 0.78).

Statistical analysis of the diameters measured from each regenerated stem at the end of the 60 day experiment (Fig. 4) showed that Rodley had significantly larger diameters than Huddersfield and Burnley when considering one and two node rhizome fragments (ANOVA: 1 node: *p* = 0.006, *F* = 6.51, 2 nodes: *p* = 0.007, *F* = 6.23) but no difference between node categories within sites. Full crowns showed no significant difference between sites (*p* = 0.190, *F* = 177, Fig. 4). Stems that originated from full crowns had a significantly larger diameter than stems from any other category across all three sites (Fig. 4).

**Figure 5:**
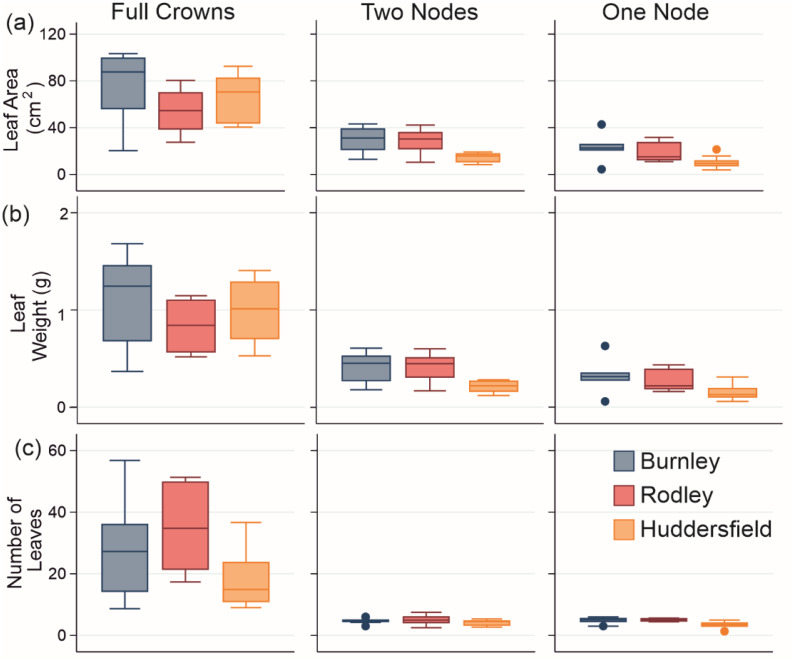
Leaf growth data from genenerated material from all sites. (A) Leaf area, (B) Leaf fresh weight and (C) Number of leaves on stems. Columns show size categories: (i) full crowns; (ii) two nodes; (iii) one node. Colours refer to sites (Burnley in blue; Rodley in red and Huddersfield in yellow) and colour tones refer to size of plant material (darkest shades for full crown, medium shades for two node rhizome fragments and lightest tone for one node rhizome fragments). Boxes represent 75th (top of box) percentile to the 25th (bottom of box) percentile of the data with the median represented by the line between the two. Data ranges are represented by the whiskers.

The maximum number of leaves that grew from a single stem was 174 from a stem that grew from a Burnley crown (Supplemental Information). The average number of leaves from crown regeneration was 28 from Burnley, 35 from Rodley and 16 from Huddersfield. Stems that regenerated from Huddersfield full crowns grew significantly fewer leaves than Rodley (ANOVA: *p* = 0.039, *F* = 3.72). Huddersfield also exhibited significantly fewer leaves (average = 3) from stems regenerating from one node fragments than the other sites (average = 5) (*p* = 0.002, *F* = 7.98), whilst two node fragments experienced no significant difference between all sites (*p* = 0.264, *F* = 1.41), with an average of five from Burnley and Rodley and four from Huddersfield (Fig. 5).

Leaf area analysis showed Burnley had significantly larger leaves than Rodley from full crowns (ANOVA: *p* = 0.107, *F* = 2.45; different Fisher’s groupings) and showed that Huddersfield leaves sampled from stems growing from 1 and 2 node fragments were significantly smaller than leaves from Burnley or Rodley (one node: Kruskal-Wallis, *p* = 0.014, *H* = 8.55; two nodes: ANOVA, *p* = 0.003, *F* = 7.63). When comparing the leaf area between the size of planted material within each site, full crowns always showed significantly larger leaf areas (Burnley: Kruskal-Wallis, *p* < 0.001, *H* = 13.11, Rodley: ANOVA, *p* < 0.001, *F* = 14.31, Huddersfield: *p* < 0.001, *F* = 57.86) than rhizome fragments (Fig. 5).

Leaf fresh weight analysis revealed no significant difference between sites for full crowns (Kruskal-Wallis: *p* = 0.115, *H* = 4.33) and Huddersfield leaves sampled from stems growing from one or two node fragments were significantly lighter than those from Burnley and Rodley (one node, *p* = 0.022, *H* = 7.64, two nodes: ANOVA, *p* = 0.003, *F* = 7.4). When considering individual sites and size categories, full crowns produced significantly heavier leaves than rhizome fragments (Burnley: Kruskal-Wallis, *p* = 0.001, *H* = 15.2, Rodley: Kruskal-Wallis, *p* < 0.001, *H* = 16.26, Huddersfield: ANOVA, *p* < 0.001, *F* = 55.63; Fig. 5). Only Rodley showed a significant difference between one and two node rhizome fragments regarding leaf weight with two node fragments producing heavier leaves than one node rhizome fragments (T-Test: *p* = 0.045, *T* = 2.22).

### Rhizome growth

**Figure 6:**
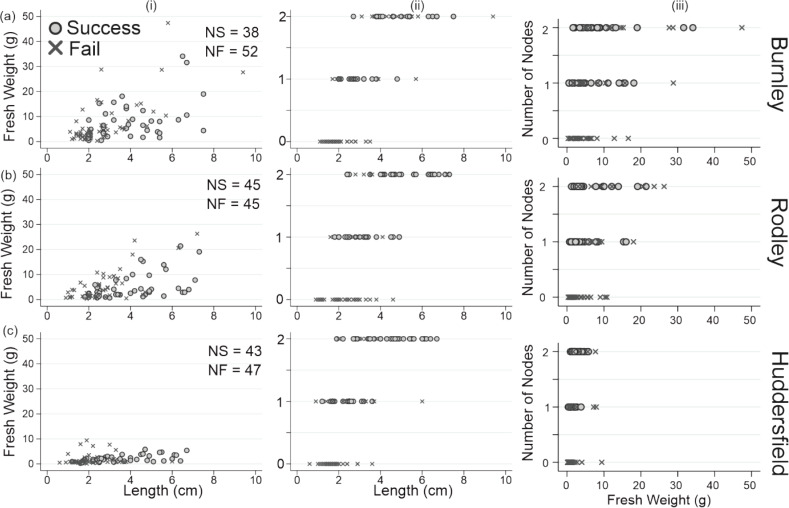
Successful and failed emergence across all rhizome fragment node categories for each site. (A) Fresh weight (g); (B) fresh weight (g); (C) fresh weight (g); (i) length v weight; (ii) length v number of nodes; weight v number of nodes. Top row: Burnley; Middle row: Rodley; Bottom row: Huddesfield; NS, number of successful emergences; NF, number of failed emergences.

Figure 6 shows successful regeneration according to initial weights and lengths of the rhizome fragments from all node categories. There is an increase in successful regeneration with increasing number of nodes that is not apparent when considering weight. Although there is an increase in regeneration success with increasing length, this only applies when the longer fragment also had more nodes. This suggests that regeneration success is most associated with number of nodes in *R. japonica* rhizomes. It also shows that fragments with no nodes did not have any emergences or regenerations regardless of fragment weight or length.

### Desiccation

**Figure 7:**
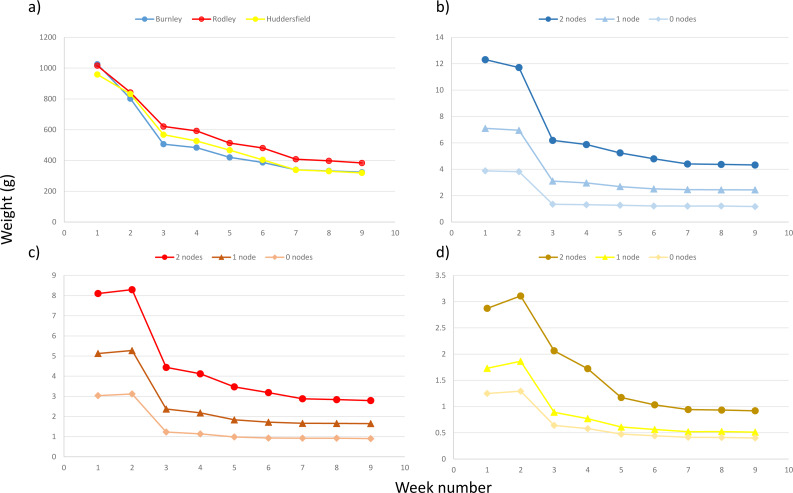
Decrease in dry weight with air drying for all fragment sizes from all sites. (A) Average for all crowns per site; (B) average for rhizome fragments from Burnley; (C) average for rhizome fragments from Rodley, Leeds; (D) average for rhizome fragments from Huddersfield. For b –d darkest colour tones with circle symbol shape = two node fragments; middle colour tones with triangle symbol shape = one node fragments; lightest colour tone with diamond symbol shape = zero node fragments.

Figure 7 shows the decrease in mass recorded after plant fragments were removed from the soil at the end of the 60-day experiment and air dried. By the 33rd day of air drying all crowns were at (or close to) their dry weight. No more than 10% of the remaining mass was lost between days 35 and 38, indicating that virtually all moisture was gone from the plant fragments. When exposed to air, plant material can potentially take up small amounts of moisture, so this was considered sufficient time to record how long it took the plant fragments to dry. For rhizome fragments with one or two nodes, there was little if any change in weight after day 11 and rhizome fragments with no nodes reached dry weight between day 11 and day 21.

All plant fragments were replanted after 38 days of air drying and treated the same way as throughout the growth experiment in relation to water and nutrient supplementation for 60 days. Regardless of size or plant fragment type, no growth was recorded from any crown or rhizome during the 60 days that they were monitored after being replanted post air drying.

## Discussion

### Growth

#### Herbicide treatment

Herbicide treatment is well described as a means of reducing growth of *R. japonica* over several years when applied correctly (Jones et al., 2018; Jones & Eastwood, 2019). The findings of this study, that there was no significant difference between crowns sampled from treated and untreated sites for regenerated stem diameter, maximum stem height and maximum growth increments, were therefore surprising because they indicate that the crowns were all equally able to produce new growth, regardless of treatment history. The treatment for the Rodley and Burnley sites included one application of a glyphosate based herbicide annually in autumn over two years. However, the observation of significant aboveground growth at these sites even after two years of treatment, which is not typical for stands of this size/maturity if best practice has been followed, suggests incorrectly applied herbicide rather than evidence for ineffective herbicide. This suggests that the active ingredient of the herbicide did not reach the underground biomass in sufficient quantity (Hagen & Dunwiddie, 2008), highlighting the importance of ensuring that herbicide is applied correctly to ensure that maximum benefits from the treatment are achieved.

Another surprising result was that both leaf area and fresh weight were significantly greater for leaves grown from rhizome fragments in the treated sites than at the untreated site at Huddersfield. Beerling (1990) showed that within two weeks of one application of glyphosate the proportion of leaves to stems (in % of total dry weight including leaves and stems) decreases below 10%. Within 14 weeks this proportion returned to its original state and when measuring Leaf Area Ratio (LAR; exposed leaf area per unit dry weight of the plant), it increased above that of the control plants with no treatment (Beerling, 1990). Alternatively, because the rhizome fragments for both two-nodes (Kruskal Wallis; *p* < 0.0001; *H* = 28.15; Supplemental Information) and one-nodes (Kruskal Wallis; *p* < 0.0001; *H* = 25.44; Supplemental Information) were significantly lighter in Huddersfield (the untreated site) than at either Burnley or Rodley, this may provide an explanation for the reduced number and size of leaves produced by these rhizomes but should not affect emergence, which was the focus of the study. This would require additional sampling to confirm that is beyond the scope of the current study. These findings show the complexity in the relationship between biomass, leaf and plant productivity with the application of herbicides and requires further investigation as leaf size is relevant to herbicide application success –the smaller the leaves, the less herbicide they can take up (Norsworthy, Burgos & Oliver, 2001).

An important finding from this study is that 11.5% of new stems (38 stems), emerged after 30 days of the start of the experiment (Fig. 3) and two emergences occurred on the final day (day 60), which suggests that further emergences could occur past the 60 day period. Although dormancy is an accepted trait of *R. japonica* and its rhizomes (Phlorum, 2019), this research shows that it should be a more considered factor regarding methodologies and lengths of experiments. Thirty days is far from sufficient when investigating emergence or regeneration for this species and likely for other rhizomatous species (e.g., 30 days: Bímová, Mandák & Pyšek, 2003; Pyšek et al., 2003, and 28 days: Francis, Riley & Hoggart, 2008). Although dormancy was not within the scope of this research, Fig. 3 shows that the rhizome categories were dominant in new emergences after day 21, previous to which, full crowns had the majority of emergences. This pattern of emergence suggests that the larger the disturbance (cutting to smaller fragments), the longer the lag time before regeneration but this requires further work to fully investigate. Francis, Riley & Hoggart (2008) showed that only three of 14 replicates of 10 cm rhizome fragments regenerated after 19 days. However, in the current study, only 40.6% (65/160) of the total stems had emerged from rhizome fragments by day 19 and the remaining 59.4% (95/160) within the next 41 days. This should inform and encourage future studies to consider longer growing periods to allow all size categories equal opportunities to grow and may also be relevant when considering management techniques and monitoring outcomes of various management strategies.

#### Nodes

Rodley exhibited significantly larger stem diameters than Burnley and Huddersfield when considering stems growing from rhizome fragments and had significantly taller stems than Huddersfield for both rhizome categories. Previous literature is unclear about which rhizome variable most significantly impacts successful regeneration, including length (Sásik & Pavol Jr, 2006) and weight (Child & Wade, 2000). One clear finding of this experiment is that rhizome fragments without a node present, regardless of treatment history, weight or length, were unsuccessful in regeneration across all three sites. A total of 90 rhizome fragments were planted with zero nodes (30 from each site; three from each crown but from separate rhizomes) with a range of lengths from 0.6 cm to 4.6 cm and a range of weights from 0.1 g to 16.6 g, no new above or belowground biomass was observed over the 60-day growing period. Rhizome fragments with one node were the smallest node category to show successful regeneration and they did so across all three sites (Burnley = 53.3%, Rodley = 80% and Huddersfield = 60%). This finding is supported by the literature with Murrell et al. (2011) showing an 83.3% success rate for one node rhizome regeneration in their experiment. Current literature accepts that new stems can regenerate from rhizome fragments as light as 0.7 g (Child & Wade, 2000; Kidd, 2000; Bailey, Bímová & Mandák, 2009). This study has shown that fragments with lower weights can also successfully regenerate. Three rhizomes fragments weighing 0.5 g, all with one node, successfully regenerated. And one Huddersfield fragment with one node weighing 0.3 g also regenerated but died after 16 days. This study shows that 0.3 g is a viable mass for regeneration but also that the number of leaves produced and likelihood of survival past two weeks is low. The study also clearly defines the number of nodes needed for regeneration and supports the reduction of the smallest accepted mass for regeneration to 0.5 g.

There were no significant differences between fragments with one or two nodes at any of the sites for maximum height and weekly growth increments. The majority of comparisons show that the quality of growth (e.g., height, leaf number, stem diameter etc; Figs. 4 and 5) was not altered by the number of nodes on fragments. Contrastingly, the percentage of successful regeneration between the node categories showed more variation. Huddersfield rhizome fragments with two nodes had a greater percentage of regeneration (83.3%, 25/30 pots regenerated) compared to those with one node (60%, 18/30 regenerated). Regeneration from Burnley material was also greater from two nodes (66.7%) than one node (53.3%) when comparing rhizome fragments. These observations show that if a fragment has one or two nodes, this does not necessarily increase the quality of the new stem that emerges from a rhizome fragment but does increase the likelihood of regeneration. Contrastingly, regeneration from Rodley material showed no apparent pattern related to size, as full crowns (80%), two nodes (76.7%) and one node fragments (80%) all had similar percentages of successful regeneration but crowns produced more stems that were taller, thicker and bore more leaves than those from rhizome fragments.

De Waal (2001) suggested that the necessary hormones for growth and regeneration, auxin and cytokinin, can be found in the nodes of *R. japonica* stems and this study suggests the same for rhizome nodes as regeneration was observed once a single node was present but never when there were no nodes on a rhizome fragment. However, the findings also show that increasing the number of nodes and particularly increasing from rhizome fragments with one or two nodes to full crowns, increases the chance of successful regeneration. This is similar to *Pennisetum macrourum,* an African perennial grass (Harradine, 1980) and *Equisetum arvense* (Marshall, 1986; Husby, 2013) which can regenerate from one node, showing that other rhizomatous species follow similar patterns in which regeneration can occur from a single node. Additionally, Alshallash & Hatcher (2020) identified the presence of rhizome tissue as key to regeneration in two *Rumex* species.

### Desiccation

Few plants can withstand desiccation and once a constant dry weight is reached, plant material is generally considered dead. There is no particular reason to assume that *R. japonica* would be any better able to recover from desiccation than other angiosperms; however we could not find an instance of where this has been tested. No new growths emerged from the planted dry biomass from either crowns or rhizomes after 60 days air drying, which suggests that the *R. japonica* material had been killed by the period of air drying and was no longer capable of emerging or regenerating. Smaller rhizomes and rhizomes in the zero node category reached dry mass faster than larger fragments and crowns, but 30 –38 days was sufficient for even the larger plant fragments to reach constant dry weight. The desiccation experiment was conducted *via* air drying, rather than use of an oven as in other desiccation experiments, e.g., Alshallash & Hatcher (2020), to purposely simulate what might be possible for home-owners with a small to moderate infestation. The experiment suggests that if carefully removed and left to air dry for approximately 38 days, then even medium sized crowns are likely to have died and be unable to produce new emergences or regenerate. Doubling this time to 76 days would provide an extremely conservative measure and likely enhanced protection from regeneration. It is worth noting that the laboratory was warm and dry, meaning that this is likely to work better during the warmer summer months and requires a warm, dry location to facilitate air drying to kill the plant material. This requires field trials, but strongly suggests a useful methodology for dealing with small *R. japonica* infestations, particularly in residential settings. Careful removal of crowns and larger pieces of rhizome will remove the material with the highest regenerative capabilities, which may simplify subsequent herbicide treatment by reducing the potential for regrowth and the quantity of herbicide required. This research demonstrates that such material can be desiccated relatively quickly and that once desiccated the material is no longer capable of growth. Accordingly, this opens up options for reuse of desiccated plant material in log piles or dead hedge (as refuges for wildlife) or returning the material to the soil to decompose.

Given concern around the use of herbicides in general (e.g., Hasanuzzaman et al., 2020; Thiour-Mauprivez et al., 2019) and glyphosate in particular (e.g., Farina et al., 2019; Sikorski et al., 2019) and that some studies suggest herbicides may become less effective under elevated CO_2_ in the mid-term future (Waryzak et al., 2018), identifying methods of controlling invasive non-native plant species that do not rely solely on herbicides should be considered a priority. Although, it is not suggested that this would be a viable management strategy for landscape-scale infestations, desiccation could be a useful component of a holistic management strategy for smaller infestations that are more typical of residential settings.

## Conclusion

The findings of this study have potential implications for management of *R. japonica* in terms of small to mid-sized infestations, particularly in the context of urban residential gardens. The growth experiment showed that crowns have higher regenerative capacity than rhizome fragments. As such, their removal prior to herbicide treatment could reduce the potential for regrowth and reduce the quantity of herbicide or time required to achieve control. Furthermore, the less vigorous growth associated with smaller fragments, should not be considered as much of a problem in general, e.g., in residential garden settings crown and large fragment removal (followed by desiccation) could be used to successfully keep *R. japonica* in check. Secondly, this experiment shows clearly that if there are no nodes present then there is no emergence or regeneration. Thirdly, desiccation was found to be an effective means of killing *R. japonica* material of all sizes. This last finding requires field trials but strongly suggests that desiccation can be used to effectively eliminate the risk of regrowth and spread from excavated plant material, potentially reducing costs associated with disposal and providing more options for reuse. It is worth re-iterating that desiccation treated crowns and rhizomes resulted in no instances of regrowth in any treatment, which, given the well documented challenges of controlling *R. japonica*, is a significant observation.

##  Supplemental Information

10.7717/peerj.11783/supp-1 Supplemental Information 1 Raw data

## Additional Information and Declarations

### Field Study Permissions

The following information was supplied relating to field study approvals (i.e., approving body and any reference numbers): Permission to sample at Rodley and Burnley was granted by the Canal and River Trust (landowners) and permission to sample at Huddersfield was granted by the Yorkshire Wildlife Trust (landowners).

### Data Availability

The following information was supplied regarding data availability: All raw data are available in the Supplemental File.
